# Reliability and validity of the Psychiatric Inpatient Patient Experience Questionnaire – Continuous Electronic Measurement (PIPEQ-CEM)

**DOI:** 10.1186/s12913-022-08307-5

**Published:** 2022-07-11

**Authors:** Hilde Hestad Iversen, Mona Haugum, Oyvind Bjertnaes

**Affiliations:** Norwegian Institute of Public Health, PO Box 222 Skoyen, Oslo, 0213 Norway

**Keywords:** Questionnaire, Mental healthcare, Patient experiences, Validation, Psychometrics, Reliability, Validity

## Abstract

**Background:**

The increasing emphasis on patient-centred care has accelerated the demand for high-quality assessment instruments, but the development and application of measures of the quality of care provided for mental health have lagged behind other areas of medicine. The main objective of this study was to determine the psychometric properties of the Psychiatric Inpatient Patient Experience Questionnaire – Continuous Electronic Measurement (PIPEQ-CEM), which consists of large-scale measurements from a Norwegian population. The change from cross-sectional surveys to continuous measurements necessitated further validation of the instrument. The secondary objective was to develop a short version of the PIPEQ-CEM.

**Methods:**

The data included responses from the first year of continuous measurement, and included adult inpatients (age ≥ 18 years) who received specialized mental healthcare from 191 different sections in Norway (*n* = 3,249). Missing data, ceiling effects, factor structure and internal consistency levels were assessed. The short scale was developed by exploring missing items, ceiling effects, results from exploratory factor analysis (EFA) and item performance from item response theory (IRT) analyses.

**Results:**

Psychometric testing supported previous results and illustrated that the PIPEQ-CEM comprises three empirically based scales with good internal consistency, reliability and validity, and covers structure and facilities, patient-centred interactions, and outcomes. A seven-item short form was developed, which provides an efficient approach for brief yet comprehensive measurements that can be applied in the future.

**Conclusion:**

The PIPEQ-CEM can be recommended for use in future national surveys that assess patient experience with inpatient psychiatric care in Norway and in other countries with similar healthcare systems. The short form can be applied where respondent burden and cognitive load are crucial issues.

**Supplementary Information:**

The online version contains supplementary material available at 10.1186/s12913-022-08307-5.

## Background

Patient experiences represent important data when evaluating the quality of healthcare services and are essential in identifying the extent to which healthcare services are achieving their purposes. Mental disorders are common worldwide, but according to Kilbourne et al. [1], their quality of care has not increased to the same extent as that for physical conditions, and the development and application of measures of the quality of mental healthcare have lagged behind other areas of medicine. Previous reviews concluded that the quality of mental healthcare remains lower than that of other medical disciplines [2], and a fundamental shift in the mental healthcare provider–service user relationship is recommended, and should incorporate the participation and involvement of service users in their own care [3, 4].

The Norwegian Institute of Public Health (NIPH) conducts national patient-experience surveys in Norway. The Psychiatric Inpatient Patient Experience Questionnaire (PIPEQ) was originally developed and validated to measure post-discharge experiences of patients in Norway [5, 6]. A change from post-discharge to on-site data collection was implemented in 2013. The surveys had cross-sectional designs, and they included patients at different phases of their treatment. The content of the Psychiatric Inpatient Patient Experience Questionnaire – On Site (PIPEQ-OS) was adjusted to on-site data collection and updated according to the latest developments of the national program [7]. In 2019, based on a recommendation from the NIPH, the Ministry of Health and Care Services decided that from 2020, the experience surveys for inpatients receiving mental healthcare and treatment for substance dependence should be conducted as continuous electronic measurements. The measurements were still conducted on-site, but as close as possible to the time of discharge. Previously developed and validated measures were applied [7, 8]. The innovations in the patient-reported experience measure (PREM) methodology aimed to improve the use of these data both at the system level, by integrating them into a multidimensional performance evaluation system, and at the individual ward level, by supporting the adoption of patient experiences for day-by-day operational management by healthcare professionals.

The changes in the data collection model necessitated further validation of the instrument. Employees at the psychiatric institutions conducting the national surveys in Norway also raised concerns regarding the cognitive abilities and motivations of patients, and emphasized the need for a shorter questionnaire. A previous study identified a set of short generic questions chosen from the established range of instruments that covered patient experiences across a range of specialist healthcare services, including psychiatric inpatient care [9]. However, the main aim was to compare results between different healthcare services, rather than informing decision-making or evaluating operational management services.

The construction and validation of patient-reported outcome (PRO) measures have traditionally been guided by classical test theory (CTT), but item response theory (IRT) may address practical measurement problems found in health-related research that have been difficult to solve using classical methods [10]. IRT provides richer and more accurate descriptions of performance at the item and scale levels, and can increase the precision and standardization of measures while reducing burdens on the respondents [10]. Reviews of PREMs in mental healthcare have indicated that the instruments differ in scope, content and psychometric robustness [2, 11]. One of these reviews identified four studies that used statistical IRT methods as a supplement to CTT, but only to assess unidimensionality or to contribute to selecting the optimal test items to shorten the instrument and enhance its clinical utility. The psychometric validation and testing in the national survey program in Norway have until recently applied classical test theory analysis, including for PIPEQ, which is in line with most validation studies related to patient experiences in mental health care [2]. However, modern test theory has a range of advantages compared to classical test theory, including more item-level information that might be used to shorten instruments.

The main objective of the present study was to determine the data quality, validity and internal consistency reliability of the Psychiatric Inpatient Patient Experience Questionnaire – Continuous Electronic Measurement (PIPEQ-CEM), which is used for large-scale measurements in Norway. The secondary objective was to create a short version of this instrument to reduce its burden on respondents and its cognitive load by supplementing classical psychometric methods with IRT to improve how the performance of the items is understood.

## Methods

### Questionnaire development and content

The questionnaire used in the PIPEQ-CEM is based on PIPEQ and PIPEQ-OS [5, 7], where development and validation followed the standard methodology from NIPH to ensure sound psychometric properties, including a systematic review of the literature, patient interviews, consultation with an expert group and pilot testing. The PIPEQ-CEM was based on the PIPEQ-OS, but with small adjustments made related to the use of the “not applicable” response in some items. Questions considered less generic in a cross-sectional setting were now more relevant, since all patients were close to discharge and the PIPEQ-CEM was adapted to the current developments in data collection. Additional File 1 contains the questionnaire.

The PIPEQ-CEM comprises 46 closed-ended items, with 21 items addressing inpatient experiences with their care scored using a 5-point response format and considered relevant for inclusion in the initial factor analyses. The final question was open-ended and probed further comments on experiences with their respective psychiatric institution. The questionnaire was divided into different sections addressing waiting time and admission, therapists and staff, involvement, information, environment and activities, negative events or incidents, enablement, follow-up on physical health, overall institution assessments, municipal help, overall healthcare service assessments and background questions. The background questions included sociodemographic variables and questions about stay duration, admission type, main reason for admission, admission necessity, previous admissions, self-perceived mental and physical health, overall current state, and coercion and offensive or incorrect treatment by personnel.

Most of the items on experience were scored on a 5-point response scale ranging from 1 (“not at all”) to 5 (“to a very large extent”). The 5-point response scale was chosen by the NIPH to be consistently applied in the surveys, making it possible to compare over time, and to some extent between different healthcare user groups [7–9, 12–14]. Most questionnaires addressing patient experiences have used items with scales where each point has a descriptor [15].

### Data collection

Continuous measurements were obtained from adult inpatients (age ≥ 18 years) who received specialized mental healthcare.

In Norway specialized mental healthcare is organised under 4 health regions, with underlying hospital trusts. All public and private residential institutions with a contract with regional health authorities were included in the surveys. The surveys were carried out at the lowest care level in psychiatric care. The sections are in some instances institutions, in other instances they are units organized under a hospital. The NIPH gathered regional contact persons to help compile the institution lists and establish contact persons at the health authority, health trust, institutional, department and section levels. A project manager was assigned to each participating unit, with tasks including disseminating information to the patients and employees, distributing login information to patients, and reporting the progress of the survey to the NIPH.

The data applied in the present study included responses from the first year of continuous measurements from psychiatric institutions in Norway, from the 1 January 2020 to 31 December 2020, and included 191 different sections and 3,249 patient responses. Table 1 lists the characteristics of these respondents: 58.3% were female, 63.8% were 18–44 years old, 32.4% had been educated to a university or college level, 29.1% were married or living with a partner, 89.3% were born in Norway, and 43.1% had three or more previous admissions. The duration of stay for 25.8% of participants was more than 4 weeks. The previous-week mental health was considered to be very poor or rather poor by 75.8% of them; the corresponding result at the response time was 34.1%. The general conditions at response time were very poor or rather poor for 19.4% of them, while 17.9% considered their physical health to be excellent or very good.

**Table 1:** Respondent characteristics (*n* = 3249)

	n	%
Sex		
Female	1865	58.3
Male	1336	41.7
Age, years		
18–24	604	18.8
25–44	1445	45.0
45–66	1007	31.3
≥ 67	158	4.9
Education		
Primary school	591	18.4
Secondary school	1575	49.2
University or college	1038	32.4
Married or living with a partner		
Yes	935	29.1
No	2276	70.9
Country/region of birth		
Norway	2873	89.3
Nordic country other than Norway	56	1.7
Western Europe other than a Nordic country	33	1.0
Eastern Europe in EU	52	1.6
Eastern Europe not in the EU	18	0.6
Africa	51	1.6
Asia (including Turkey)	98	3.0
North America	9	0.3
South America or Central America	22	0.7
Oceania	6	0.2
Previous admissions		
0	926	28.8
1	558	17.4
2	328	10.1
3–5	508	15.6
> 5	893	27.5
Length of stay at this institution		
< 1 day	51	1.6
1 or 2 days	136	4.2
3–7 days	773	23.9
1–4 weeks	1439	44.5
1–6 months	762	23.6
> 6 months	71	2.2
Self-perceived mental health the week prior to admission		
Very poor	1458	45.2
Rather poor	986	30.6
Both	529	16.4
Rather good	180	5.6
Very good	73	2.3
Self-perceived mental health		
Very poor	303	9.5
Rather poor	787	24.6
Both	1306	40.7
Rather good	628	19.6
Very good	181	5.6
General condition today		
Very poor	191	5.9
Rather poor	435	13.5
Both	1251	38.9
Rather good	1063	33.1
Very good	272	8.5
Self-perceived physical health		
Excellent	150	4.7
Very good	424	13.2
Good	1136	35.4
Rather good	992	30.9
Poor	508	15.8

### Statistical analysis

Missing data and ceiling effects were assessed. Items with missing data or > 20% “not applicable” responses were excluded [7, 8]. The cut-off criterion for ceiling effects was 50%; that is, an item was considered acceptable if fewer than 50% of the respondents chose the most-favourable response option [16].

We operate in line with the international scientific literature where patient experience predominantly is analysed as a reflective construct [2, 17], and we applied classical test theory to obtain information about the properties of the instrument. Major changes in data collection from cross-sectional surveys to continuous measurements necessitated a revalidation of the instrument. When the surveys were carried out with a cross-sectional design, patients were asked for their experiences at different phases of their treatment, and they responded on a paper questionnaire. In the current measurements, patients are invited to reply to an electronic version of the questionnaire a few days before discharge. Therefore, EFAs were conducted to establish the underlying structure on empirical and theoretical grounds. EFAs were supplemented with IRT analyses because the latter provides a much richer description of the performance of each item, which was useful during refinement to ensure that the best items were selected for the short form [10]. Consequently, the use of IRT was instrumentally justified by the aim of creating a short form of the questionnaire.

EFA with principal-axis factoring and oblique rotation was conducted to identify the internal structures of the PIPEQ-CEM. Missing data were accounted for using listwise deletion. The number of extracted factors was decided based on an eigenvalue criterion of > 1. The analysis and resulting indicators were based on statistical testing and psychometrics, and also theoretical considerations. Regarding theoretical considerations, the outcome items were separated from the process and structure items in the EFAs, and were consistent with those in with previous psychometric testing of the national survey program. The internal consistency reliability of each indicator was assessed using the total correlation coefficient and Cronbach’s alpha for the items.

We conducted two IRT analyses to further evaluate item performance. Firstly, we applied IRT analysis to the items identified in the EFA that addressed patient-centred interactions and outcomes. The structure and facilities scale included structural components of healthcare also assumed to be fundamental in achieving high quality care, but not assumed to be theoretically similar enough to be combined with the items from the patient-centred interactions and outcomes scales in the analysis. Unidimensionality is assumed in the IRT, and that responses to the items depend on a single underlying dimension consistent across all items in the test. The number of items (four) made the structure and facilities scale scale less suitable for IRT, but we nevertheless decided to apply IRT analysis to the structure and facilities scale to obtain more information about item performance.

The instrument had polytomous response options, and the generalized partial credit model (GPCM) was used. The GPCM is one of the most flexible polytomous IRT models because it has fewer assumptions than for example the partial credit, rating scale or Bock’s nominal models, and allows for separate discrimination parameters and separate category response parameters to be estimated for each item [10]. The model has the advantage of allowing slope parameters to vary across items, and threshold parameters between response categories give an indication on the response options’ locations along the latent construction continuum. As a result of this flexibility, this model is more likely to fit data generated from patient-reported outcome measures. Missing data were omitted from the analysis. Item performance was based on assessments of item discrimination (a) and item location or difficulty (b), and also on the S − χ^2^ item-fit statistic. For the latter, the null hypothesis was that the item fits well, and a significant result indicates it was a poor fit [10]. However, these fit indices are sensitive to sample size, and even negligible differences can produce a result indicating a poor fit in large samples.

A shorter version of the instrument was identified by assessing item missing, ceiling effects, EFA results, and item performance from the IRT analyses. We aimed to secure content coverage and selected the best-performing items for each of the scales. The concordance between the long and short versions of the PIPEQ-CEM scales was assessed using intraclass correlation coefficients (ICCs). ICCs were calculated based on absolute agreement with a two-way mixed-effects model. Statistical analyses were performed using SPSS version 25.0 and the R statistics software version 4.0.2 (package mirt).

## Results

The proportions of missing data, “not applicable/do not know” responses, mean values and ceiling effects for the 21 items relevant for inclusion in the psychometric testing are listed in Table 2. The results indicate that 20 of the 21 items in the PIPEQ-CEM had low proportions of missing/not-applicable responses (< 20%); an exception was the cooperation-with-relatives item (25.5%). Only the incorrect-treatment item did not comply with the ceiling-effect criterion (< 50% in the most-positive response option), which was excluded in further analyses.

**Table 2:** Item descriptions

	n	*Missing (%)*	*Not applicable (%)*	*Mean*^a^	*Ceiling (%)*
4. Was the way you were welcomed to the institution satisfactory?	3199	0.5	1.0	4.17	43.3
8. Have you had enough time for discussions and contact with the therapists/staff?^b^	3204	0.7	0.6	3.91	28.2
9. Do you find that the therapists/staff have understood your situation?^b^	3210	0.6	0.6	3.90	31.2
10. Have you had the opportunity to tell the therapists/staff about important aspects of your condition?	3195	0.7	0.9	3.98	31.1
11. Do you find that the therapists/staff have cooperated well with your relatives?	2422	0.6	24.9	3.60	23.6
12. Do you find that the therapists/staff have prepared you for the time after discharge?	3097	0.7	4.0	3.49	17.4
13. Do you find that the treatment has been adapted to your situation?^b^	3183	0.7	1.3	3.74	23.6
14. Have you had influence on your choice of treatment?	3083	1.0	4.1	3.36	15.6
15. Have you had influence on your choice of medication?	2839	0.8	11.8	3.38	19.7
17. Has the institution provided you with sufficient information on your mental health/diagnosis?	2976	0.8	7.6	3.48	19.0
18. Has the institution provided you with sufficient information on the treatment options available to you?	3050	0.7	5.4	3.36	15.3
19. Have you felt safe at the institution?^b^	3199	0.7	0.8	4.23	46.7
20. Has the range of activities available at the institution been satisfactory?^b^	3038	0.9	5.6	3.52	19.8
21. Have the meals at the institution been satisfactory?	3151	1.0	2.0	3.92	34.1
22. Have you been satisfied with the level of privacy available?	3163	1.0	1.7	3.97	33.5
24. Do you believe that you have been incorrectly treated in any way while at the institution (according to your own judgement)?	3151	1.0	2.1	4.33	61.4
25. Are the help and the treatment you are receiving at the institution helping you better understand your mental health issues?^b^	3070	1.1	4.4	3.52	19.5
26. Are the help and the treatment you are receiving at the institution helping you better cope with your mental health issues?	3092	1.1	3.8	3.44	15.2
27. Are the help and the treatment you are receiving at the institution giving you confidence that life will be better after discharge?	3110	1.1	3.1	3.38	16.7
28. Overall, have the help and the treatment you have received at the institution been satisfactory?^b^	3213	1.1	0.0	3.83	25.0
29. Overall, to what extent have you benefitted from the treatment at the institution?^c^	3202	1.4	0.0	3.58	20.4

^a^ Most items were scored on a 5-point response scale ranging from 1 (“not at all”) to 5 (“to a very large extent”)

^b^ Items finally selected for the short form of the instrument

^c^ Item scored on a 5-point response scale ranging from 1 (“no benefit”) to 5 (“very large benefit”)

The remaining 19 items were included in the EFAs. The first EFA included the 14 items on structure and process, which yielded 2 factors. None of the items were excluded due to a cross-factor loading of > 0.30, or a loading on the same factor of < 0.40. The results suggested that two factors with an eigenvalue of > 1 accounted for 55.5% of the total variance: (i) structure and facilities and (ii) patient-centred interactions (Table 3). The second factor analysis included the five items on outcomes and produced one factor with an eigenvalue of > 1 that explained 75.1% of the variance. Table 3 lists the item-total correlations for the final three scales, which were acceptable and ranged from 0.43 to 0.83. All scales met the criterion of 0.70–0.90 for Cronbach’s alpha, which ranged from 0.76 to 0.92. Omission of items was found to not increase the Cronbach’s alpha values.

**Table 3:** Factor loadings and reliability statistics

	Factor loadin*g*^a^	Corrected item-total correlation	Cronbach’s alpha	Cronbach’s alpha if item deleted
**Structure and facilities**			0.76	
4. Was the way you were welcomed to the institution satisfactory?	0.58	0.54		0.71
19. Have you felt safe at the institution?	0.71	0.58		0.69
20. Has the range of activities available at the institution been satisfactory?	0.40	0.50		0.72
21. Have the meals at the institution been satisfactory?	0.51	0.43		0.75
22. Have you been satisfied with the level of privacy available?	0.75	0.57		0.69
**Patient-centred interactions**			0.90	
8. Have you had enough time for discussions and contact with the therapists/staff?	0.57	0.68		0.89
9. Do you find that the therapists/staff have understood your situation?	0.56	0.76		0.88
10. Have you had the opportunity to tell the therapists/staff about important aspects of your condition?	0.45	0.61		0.89
12. Do you find that the therapists/staff have prepared you for the time after discharge?	0.82	0.67		0.89
13. Do you find that the treatment has been adapted to your situation?	0.63	0.77		0.88
14. Have you had influence on your choice of treatment?	0.65	0.67		0.89
15. Have you had influence on your choice of medication?	0.46	0.49		0.90
17. Has the institution provided you with sufficient information on your mental health/diagnosis?	0.78	0.70		0.89
18. Has the institution provided you with sufficient information on the treatment options available to you?	0.85	0.70		0.89
**Outcomes**			0.92	
25. Are the help and the treatment you are receiving at the institution helping you better understand your mental health issues?	0.82	0.78		0.90
26. Are the help and the treatment you are receiving at the institution helping you better cope with your mental health issues?	0.87	0.83		0.89
27. Are the help and the treatment you are receiving at the institution giving you confidence that life will be better after discharge?	0.81	0.77		0.90
28. Overall, have the help and the treatment you have received at the institution been satisfactory?	0.81	0.77		0.90
29. Overall, to what extent have you benefitted from the treatment at the institution?	0.83	0.79		0.90

^a^ Separate factor analysis for outcome items

IRT analysis was applied to the items identified in the EFA, but the results showed that items 15 (“Have you had influence on your choice of medication?”) and 21 (“Have the meals at the institution been satisfactory?”) had markedly lower factor loadings than the other items (< 0.40). The EFA results indicated that these items also had the lowest corrected item-total correlations, and they were subsequently excluded. Table 4 lists the results of the IRT analysis on the PIPEQ-CEM, and the scales of patient-centred interactions and outcomes. The S − χ^2^ statistic of the 13 items indicated that all but 4 of the items had adequate performance, the exceptions being items 10 (“Have you had the opportunity to tell the therapists/staff about important aspects of your condition?”) (*p* < 0.001), 12 (“Do you find that the therapists/staff have prepared you for the time after discharge?”) (*p* = 0.011), 14 (“Have you had influence on your choice of treatment?”) (*p* < 0.001) and 27 (“Are the help and treatment you are receiving at the institution giving you confidence that life will be better after discharge?”) (*p* = 0.005). The values of the item discrimination parameters varied from 1.09 for item 14 (“Have you had influence on your choice of treatment?”) to 2.98 for item 28 (“Overall, have the help and the treatment you have received at the institution been satisfactory?”) (Table 4). None of the discrimination parameters exceeded 4.0.

**Table 4:** Parameter estimates from item response theory analysis according to the Psychiatric Inpatient Patient Experience Questionnaire – Continuous Electronic Measurement scales (*n* = 1772)

	a	b1	b2	b3	b4	S–χ^2^	p
**Patient-centred interactions**							
8. Have you had enough time for discussions and contact with the therapists/staff?	1.66	–2.33	–1.93	–0.76	0.69	98.48	0.079
9. Do you find that the therapists/staff have understood your situation?	2.32	–2.11	–1.63	–0.61	0.51	75.69	0.300
10. Have you had the opportunity to tell the therapists/staff about important aspects of your condition?	1.17	–2.48	–2.36	–0.99	0.61	146.48	< 0.001
12. Do you find that the therapists/staff have prepared you for the time after discharge?	1.34	–2.07	–1.35	–0.17	1.19	130.67	0.011
13. Do you find that the treatment has been adapted to your situation?	2.68	–1.93	–1.49	–0.39	0.80	73.27	0.340
14. Have you had influence on your choice of treatment?	1.09	–1.73	–1.36	0.15	1.47	165.81	< 0.001
17. Has the institution provided you with sufficient information on your mental health/diagnosis?	1.42	–1.80	–1.28	–0.12	1.10	117.46	0.059
18. Has the institution provided you with sufficient information on the treatment options available to you?	1.30	–1.84	–1.26	0.01	1.26	108.45	0.201
**Outcomes**							
25. Are the help and the treatment you are receiving at the institution helping you better understand your mental health issues?	2.42	–1.80	–1.23	–0.14	0.95	80.87	0.359
26. Are the help and the treatment you are receiving at the institution helping you better cope with your mental health issues?	2.35	–1.70	–1.30	0.03	1.18	70.74	0.735
27. Are the help and the treatment you are receiving at the institution giving you confidence that life will be better after discharge?	1.66	–1.59	–1.38	0.12	1.08	127.21	0.005
28. Overall, have the help and the treatment you have received at the institution been satisfactory?	2.98	–2.04	–1.54	–0.55	0.74	75.96	0.127
29. Overall, to what extent have you benefitted from the treatment at the institution?	2.18	–2.00	–1.46	–0.13	0.91	84.16	0.244
**Value ranges**	**1.09 to 2.98**	–**1.59 to** –**2.48**	–**1.23 to** –**2.36**	–**0.99 to 0.15**	**0.51 to 1.47**		
**Structure and facilities**							
4. Was the way you were welcomed to the institution satisfactory?	1.34	–1.90	–2.09	–1.29	0.11	57.27	< 0.001
19. Have you felt safe at the institution?	1.86	–2.20	–2.04	–1.15	0.07	27.04	0.104
20. Has the range of activities available at the institution been satisfactory?	0.89	–2.30	–1.68	–0.05	1.21	31.43	0.113
22. Have you been satisfied with the level of privacy available?	1.35	–1.94	–1.92	–0.87	0.58	33.54	0.041
**Value ranges**	**0.89 to 1.86**	–**1.90 to** –**2.30**	–**1.68 to** –**2.09**	–**0.05 to** –**1.29**	**0.07 to 1.21**		

^a^ Generalized partial credit model. a: discrimination; b1–b4: thresholds. S–χ^2^: item-fit statistic, with *p* < 0.05 indicating a lack of fit

**Fig. 1:**
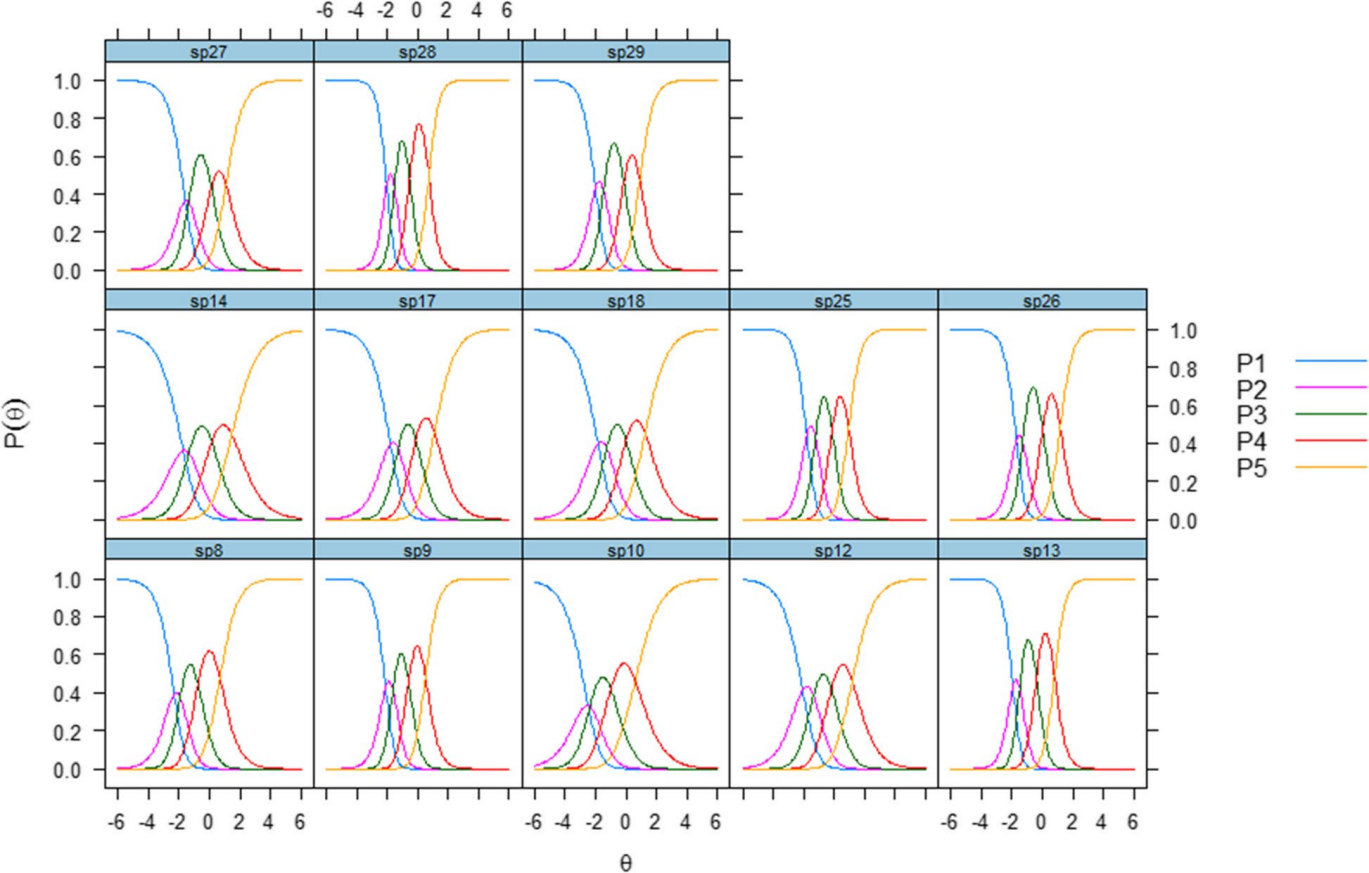
Categorical response curves of the Psychiatric Inpatient Patient Experience Questionnaire – Continuous Electronic Measurement scales, patient-centred interactions and outcomes scales

Item category thresholds varied between items, but were mostly concentrated below or around the average, indicating that item measurements were the most accurate at the lower and middle ends of the scale. The threshold of the first item varied from –1.59 to –2.48, the second from –1.23 to –2.36, the third from –0.99 to 0.15 and the fourth from 0.51 to 1.47. The categorical response curves (CRCs) in Fig. 1 indicate that the second response category had questionable values for some items, particularly 10 and 27.

The goal of scale refinement was to create a shorter scale for making meaningful interpretations in differences or changes over time between groups, and items were selected to secure an even distribution across various locations. Seven items were selected after assessing psychometric results, missing items and ceiling effects, which were sorted according to the three scales to secure coverage of patient-centred interactions, structure and facilities, and outcomes.

**Fig. 2:**
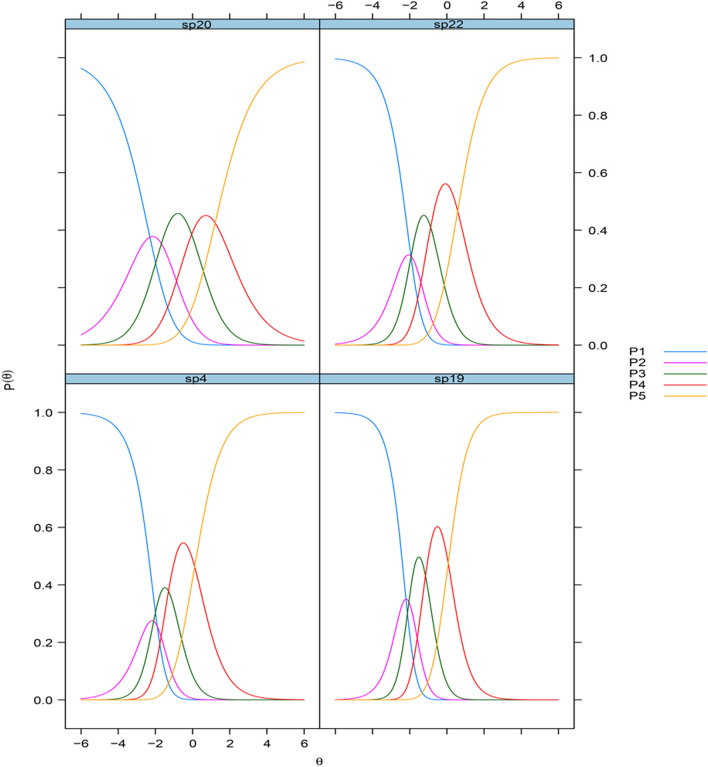
Categorical response curves of the Psychiatric Inpatient Patient Experience Questionnaire – Continuous Electronic Measurement scales, structure and facilities scale

The EFA of the structure and facilities scale revealed that the factor loadings were highest for items 22 (“Have you been satisfied with the level of privacy available?”) and 19 (“Have you felt safe at the institution?”) (Table 3). However, items 19 and 4 (“Was the way you were welcomed to the institution satisfactory?”) had higher ceiling effects than the other items within the scale (Table 2), which restricts their usefulness as measures for detecting changes or describing differences. We decided to use IRT to further aid item selection. The S − χ^2^ statistic indicated adequate performance for items 19 and 20 (“Has the range of activities available at the institution been satisfactory?”) (Table 4). Item 19 had the best discriminative ability, and item 20 measured at a higher location to the latent construct than the other items. We therefore selected items 19 and 20 for the short form of the instrument. The CRCs for the four items are shown in Fig. 2.

Regarding patient-centred interactions, items 13 (“Do you find that the treatment has been adapted to your situation?”) and 9 (“Do you find that the therapists/staff have understood your situation?”) were the items with the best discriminative abilities, with slope estimates of 2.68 and 2.32, respectively (Table 4). Item 8 (“Have you had enough time for discussions and contact with the therapists/staff?”) was also included to secure coverage of the accessibility content. Examining the information function curves further aided the selection. The CRCs indicated that the response categories of these three items covered a wide range of theta (Fig. 1). All items had lower levels of missing data, but slightly higher ceiling effects. The S − χ2 statistic shows adequate performance (non-significant results).

Regarding outcomes, item 28 (“Overall, have the help and the treatment you have received at the institution been satisfactory?”) had the best discriminative ability, followed by item 25 (“Are the help and the treatment you are receiving at the institution helping you better understand your mental health issues?”). Both items had a strong connection to the latent construct, and low levels of missing data. Results from the CRCs indicated distinct peaks for all response options that cover a wide range of theta (Fig. 1). The S − χ^2^ statistic indicated adequate performance for both of these items.

We also performed IRT analyses, with each of the two full scales measuring patient-centred interactions and outcomes independently. The results corresponded with those from the analysis including all 13 items.

The ICC between the 17-item PIPEQ-CEM scale and its 7-item short form was 0.98 (*p* < 0.001). The ICCs between the full structure and facilities, patient-centred interactions, and outcomes scales, and the selected items were 0.94 (*p* < 0.001), 0.95 (*p* < 0.001) and 0.97 (*p* < 0.001), respectively.

## Discussion

The present study had two purposes: (i) to determine the data quality, validity and internal consistency reliability of the PIPEQ-CEM used for national measurements in Norway, and (ii) to create a short version of the instrument in order to reduce the burden on respondents and the cognitive load.

The psychometric testing produced good evidence for data quality, internal consistency and construct validity. The PIPEQ-CEM was originally developed using a standardized and comprehensive process, but was adapted according to developments in data collection procedures [5–7]. The EFA results strengthened previous results and indicated that the PIPEQ-CEM discriminates between different aspects of experiences, with the following three scales: (i) structure and facilities, (ii) patient-centred interactions, and (iii) outcomes. The scales had excellent psychometric properties, and the PIPEQ-CEM was also considered relevant as a basis for identifying quality indicators. As recommended by Kilbourne et al. [1], mental healthcare quality measures need to be validated across the Donabedian spectrum, involving structure, process and outcome.

The literature review revealed a lack of similar studies, which makes it difficult to compare our results with others. A previous systematic review indicated that the most salient experiences of mental health inpatients that inform the provisions of high-quality services are high-quality relationships; averting negative experiences of coercion; a healthy, safe and enabling physical and social environment; and authentic experiences of patient-centred care [18]. Another recent review identified 75 PREMs available for mental healthcare; while 24 were designed for inpatient and residential settings, the measures differed in scope, content and psychometric robustness [2]. The most-represented dimensions were interpersonal relationships, respect and dignity, access and coordination of care, drug therapy, information, psychological care, and the care environment, which are also included in the PIPEQ-CEM. Another previous national study using the PIPEQ-OS assessed the importance of different types of patient-reported predictors for outcome assessments for mental health inpatients. The results indicated that the most important structure and process variables for patient outcome assessments were related to patient-centred interactions [19].

The relatively small proportion of “not applicable” responses and the low percentage of omitted answers suggest good acceptability and indicate that the questions are relevant to most patients. However, one of the major disadvantages of the PIPEQ-CEM reported by employees at the psychiatric institutions was the burden associated with completing it. Concerns have been raised regarding the cognitive abilities and motivation of patients, and employees have emphasized the need for a shorter questionnaire that is appropriate for patients with a wide range of literacy levels. The present study identified a seven-item short form that provides a uniquely efficient approach for brief and comprehensive measurements that can be applied in the future. The short form includes questions related to if the treatment is adapted to the situation of the patient, if the therapists/staff understand the patient’s situation, if the patient have enough time for discussions and contact with the therapists/staff, feels safe, considers the activities to be satisfactory, if the help and treatment contribute to improving their understanding of mental health issues, and if the help and treatment are satisfactory overall. The present results illustrate the detailed information that can be obtained on an instrument using a combination of EFA and IRT. Some information from the approaches overlapped, providing triangulated evidence of item quality, while other information was unique to each method. IRT provided item-level detail that informed the revising of the scale, and there was a strong correlation between full and short versions.

The national patient-experience surveys in Norway aim to systematically measure user experiences with healthcare structures and processes of care, as a basis for quality improvement, healthcare service management, free patient choice and public accountability. Previous studies have indicated that two barriers to using patient survey results include delays in disseminating results and a lack of sufficiently specific information at specific levels of healthcare [20–22]. The PIPEQ-CEM results in Norway are published only weeks after the reporting period, and reports are distributed to all units with a sufficient number of responses. Apart from a study protocol with continuous PREMs and patient-reported outcome measures (PROMs) for elective hip and knee arthroplasty [23], we could not find any research studies of large-scale or national programs for continuous measurements of patient healthcare experiences. PIPEQ-CEM represented a novel, feasible and time-effective approach by collecting large-scale data and rapidly reporting responses using web-based administration methods.

Patients with severe mental illness and substance use disorders are often considered vulnerable, and higher rates of mental disorders are associated with social disadvantage, especially alongside low income, low education and occupational statuses, and financial strain [24]. This population is also confronted with persistent gaps in access to and receiving mental healthcare, with major challenges including inadequate treatments and underused guidelines, healthcare variation among geographical regions, stigma and discrimination, and poor adherence to treatment by patients [1, 2, 4]. These studies demonstrate the importance of systematic measurements of patient experiences in mental healthcare. Although measuring patient experiences is an accomplishment in itself that might lead to quality improvement, it is necessary to make the right choices in designing reliable interventions to improve patient experiences. The PIPEQ-CEM provides feedback in specific areas, and the results can be used to monitor performance and identify departments where the quality should be improved from the patient perspective.

The three scales were empirically based, but it is essential that the survey tools and methods provide feedback that is sufficiently specific and can be acted on when conducting user-experience surveys. Further research should address the relevance of local quality improvement work on healthcare services, addressing specific experiences, and timely publishing and sharing of the results that are consistent with the patient experience. The short version of the instrument presented here can be used in settings where respondent burden and cognitive load are crucial issues, but further research is needed since the choices were only driven by data. Further research should involve an expert panel of patients and healthcare professionals to assess priorities.

The appraisals of a patient may differ throughout their hospital stay, and so interpreting the scales would benefit from standardized timing. However, data collection at discharge represents a more-time-consuming method. The NIPH has to establish contact at all levels, all institutions must establish new routines for data collection and the data collection would not be restricted to a specific day or week. Continuous communication between the NIPH and each institution is also needed to report on how the data collection is progressing. Moreover, it is harder to reach patients who drop out of treatment. Even though the number of patients in the surveys has been increasing over time, many patients are still not included. To obtain representative and useful data, all patients should be invited to participate. Future surveys should combine the existing on-site approach with a post-discharge approach for outpatients. The surveys are currently anonymous. Obtaining background data from the Norwegian Patient Registry would allow us to develop follow-up routines, and implement post-discharge surveys to supplement the on-site surveys, enabling non-response analysis and case-mix adjustments.

Web-based surveys have many advantages, but a major limitation is that they exclude those with poor digital literacy. The number of responses might have been larger if the patients also had the option to respond using a pen and paper. Pen-and-paper questionnaires induce complexity and resource demands and will not be available on-site, but national infrastructure might be used for future post-discharge surveys among patients not included on-site, and follow-up surveys for inpatients that responded on-site.

Previous research has concluded that personal contact in recruitment and data collection may increase the response rate, but there is some concern that on-site data collection is associated with different responses. On-site data collection might increase the number of responses and accordingly how representative the data are, but research indicates that on-site approaches result in more-favourable responses compared with mailed surveys [25–27]. We will assess this in future research, especially to identify a method to adjust for mode effects when comparing results obtained by different data collection modes.

The present study has highlighted the use of IRT as an important tool for developing and validating scales, and how its applications can provide richer and more accurate descriptions of performance at the item and scale levels, and allow fielding fewer questions to participants without a loss of measurement precision. However, single items are normally less reliable than scales [28], and the psychometric properties and relevance of the short form of the instrument require further evaluation. In future research we propose to develop item banks for domains of patient experiences and utilize Computerized adaptive testing (CAT) as an alternative to standard short-forms. CAT is based on IRT and allows the administration of a customized subset of items taking into account the patient’s level of ability for a latent trait. Thus, only the most suitable items to assess the quality of care perceived by the respondent will be administered, securing more accurate score estimates and a lower burden than standard fixed format questionnaires.

### Strengths and limitations

One strength of this study was that the domains and items were derived using a standardized, comprehensive process. Furthermore, the large national sample included responses from 70% of all inpatient units in Norway. The short version of the instrument will hopefully reduce dropout rates and improve the coverage of patients with poor cognitive skills.

A potential source of bias in this study was that response rates and background data on non-respondents were unavailable. Future data collection efforts should aim to include such information and predict hypothetical experiences of non-respondents in order to estimate the impact of response rates, and how these affect patient-experience data. Further research should compare respondents and non-respondents to assess if they have different experiences. Case-mix adjustment is important to fairly compare across different healthcare sections, and more evidence is needed on the impact of case-mix adjustment. The test–retest reliability of the questionnaire should be evaluated in order to determine both short- and long-term reliability, pending a formal test–retest assessment. Furthermore, the generalizability of the results to all inpatient departments in Norway is uncertain.

## Conclusions

The PIPEQ-CEM comprises three empirically based scales with good internal consistency reliability and validity covering structure and facilities, patient-centred interactions, and outcomes. This instrument can be recommended for use in future national surveys that assess patient experiences within inpatient psychiatric care in Norway and in other countries with similar organizations. The study developed a seven-item short form of the instrument that can be applied where respondent burden and cognitive load are crucial issues.

Further research should focus on obtaining background data and establishing models for weighting, case-mix adjustment and non-response analysis, and should explore the potential for constructing and reporting quality indicators based on the PIPEQ-CEMs in Norway.

## Supplementary Information

**Additional file 1.**

## Data Availability

The data sets generated and/or analysed during the current study are not publicly available due to the need to protect personal data but are available from the corresponding author on reasonable request.

## Abbreviations

NIPH: Norwegian Institute of Public Health
PIPEQ: Psychiatric Inpatient Patient Experience Questionnaire
PIPEQ-OS: Psychiatric Inpatient Patient Experience Questionnaire – On Site
PREM: Patient-reported experience measure
PRO: Patient-reported outcome
CTT: Classical test theory
IRT: Item response theory
PIPEQ-CEM: Psychiatric Inpatient Patient Experience Questionnaire – Continuous Electronic Measurement
EFA: Exploratory factor analysis
GPCM: Generalized partial credit model
ICC: Intraclass correlation coefficients
CRC: Categorical response curves
PROM: Patient-reported outcome measure
