# Supplementary material for: Reliability and validity of the Psychiatric Inpatient Patient Experience Questionnaire – Continuous Electronic Measurement (PIPEQ-CEM)
Source: BMC Health Serv Res. 2022 Jul 11;22:897. doi: 10.1186/s12913-022-08307-5 (PMC9275271; doi:10.1186/s12913-022-08307-5)
Supplement: Supplementary file 1 — Additional file 1. [file 12913_2022_8307_MOESM1_ESM.pdf]

NIPH
Norwegian Institute of Public Health

What are your experiences with your inpatient stay in mental health care?

The purpose of this survey is to improve the services for patients within mental health care. We are interested in your experiences with the institution where you are now a patient.

How to fill in the form: Enter an X in the centre of the appropriate box.
Like this: ☒ Not like this: ☐

Waiting time and admission

1. How long have you been an inpatient at this institution?

- ☐ Less than 1 day
- ☐ 1-2 days
- ☐ 3-7 days
- ☐ 1-4 weeks
- ☐ 1-6 months
- ☐ More than 6 months

2. Was your admission to this institution planned in advance or an emergency admission?

- ☐ Planned
- ☐ Emergency

3. Did you have to wait for admission?

- ☐ No
- ☐ Yes, but not long
- ☐ Yes, quite long
- ☐ Yes, far too long
- ☐ *Not applicable*

4. Was the way you were welcomed to the institution satisfactory?

- ☐ Not at all
- ☐ To a small extent
- ☐ To some extent
- ☐ To a large extent
- ☐ To a very large extent
- ☐ *Not applicable*

5. Were you admitted against your will?

- ☐ Yes
- ☐ No

6. Do you find that the admission was necessary or unnecessary?

- ☐ Very unnecessary
- ☐ Somewhat unnecessary
- ☐ Neither/nor
- ☐ Somewhat necessary
- ☐ Very necessary

7. How would you describe your mental health the week prior to this admission?

- ☐ Very poor
- ☐ Quite poor
- ☐ Neither poor nor good
- ☐ Quite good
- ☐ Very good

Therapists and staff

Keep the therapists and staff at the institution in mind when you answer the following questions.

8. Have you had enough time for discussions and contact with the therapists/staff?

- ☐ Not at all
- ☐ To a small extent
- ☐ To some extent
- ☐ To a large extent
- ☐ To a very large extent
- ☐ *Not applicable*

9. Do you find that the therapists/staff have understood your situation?

- ☐ Not at all
- ☐ To a small extent
- ☐ To some extent
- ☐ To a large extent
- ☐ To a very large extent
- ☐ *Not applicable*

10. Have you had the opportunity to tell the therapists/staff about important aspects of your condition?

- ☐ Not at all
- ☐ To a small extent
- ☐ To some extent
- ☐ To a large extent
- ☐ To a very large extent
- ☐ *Not applicable*

11. Do you find that the therapists/staff have cooperated well with your relatives?

- ☐ Not at all
- ☐ To a small extent
- ☐ To some extent
- ☐ To a large extent
- ☐ To a very large extent
- ☐ *Not applicable*

12. Do you find that the therapists/staff have prepared you for the time after discharge?

- ☐ Not at all
- ☐ To a small extent
- ☐ To some extent
- ☐ To a large extent
- ☐ To a very large extent
- ☐ *Not applicable*

Involvement at the institution

13. Do you find that the treatment has been adapted to your situation?

- ☐ Not at all
- ☐ To a small extent
- ☐ To some extent
- ☐ To a large extent
- ☐ To a very large extent
- ☐ *Not applicable*

14. Have you had influence on your choice of treatment?

- ☐ Not at all
- ☐ To a small extent
- ☐ To some extent
- ☐ To a large extent
- ☐ To a very large extent
- ☐ *Not applicable*

Involvement at the institution

15. Have you had influence on your choice of medication?

- ☐ Not at all
- ☐ To a small extent
- ☐ To some extent
- ☐ To a large extent
- ☐ To a very large extent
- ☐ *Not using medication*
- ☐ *Not applicable*

16. Is the treatment during this stay voluntary or do you feel forced to receive it?

- ☐ Completely voluntary
- ☐ Somewhat voluntary
- ☐ Neither voluntary nor involuntary
- ☐ Somewhat involuntary
- ☐ Completely involuntary
- ☐ *Not applicable*

Information

17. Has the institution provided you with sufficient information on your mental health/diagnosis?

- ☐ Not at all
- ☐ To a small extent
- ☐ To some extent
- ☐ To a large extent
- ☐ To a very large extent
- ☐ *Not applicable*

18. Has the institution provided you with sufficient information on the treatment options available to you?

- ☐ Not at all
- ☐ To a small extent
- ☐ To some extent
- ☐ To a large extent
- ☐ To a very large extent
- ☐ *Not applicable*

Environment and activities

19. Have you felt safe at the institution?

- ☐ Not at all
- ☐ To a small extent
- ☐ To some extent
- ☐ To a large extent
- ☐ To a very large extent
- ☐ *Not applicable*

20. Has the range of activities available at the institution been satisfactory?

- ☐ Not at all
- ☐ To a small extent
- ☐ To some extent
- ☐ To a large extent
- ☐ To a very large extent
- ☐ *Not applicable*

Environment and activities

21. Have the meals at the institution been satisfactory?

- ☐ Not at all
- ☐ To a small extent
- ☐ To some extent
- ☐ To a large extent
- ☐ To a very large extent
- ☐ *Not applicable*

22. Have you been satisfied with the level of privacy available?

- ☐ Not at all
- ☐ To a small extent
- ☐ To some extent
- ☐ To a large extent
- ☐ To a very large extent
- ☐ *Not applicable*

Negative events/incidents

23. Have you been patronised or insulted by the therapists/staff while at the institution?

- ☐ No, never
- ☐ Yes, once
- ☐ Yes, a few times
- ☐ Yes, many times

24. Do you believe that you have been incorrectly treated in any way while at the institution (according to your own judgement)?

- ☐ Not at all
- ☐ To a small extent
- ☐ To some extent
- ☐ To a large extent
- ☐ To a very large extent
- ☐ *Not applicable*

Other assessments

25. Are the help and the treatment you are receiving at the institution helping you better *understand* your mental health issues?

- ☐ Not at all
- ☐ To a small extent
- ☐ To some extent
- ☐ To a large extent
- ☐ To a very large extent
- ☐ *Not applicable*

26. Are the help and the treatment you are receiving at the institution helping you better *cope with* your mental health issues?

- ☐ Not at all
- ☐ To a small extent
- ☐ To some extent
- ☐ To a large extent
- ☐ To a very large extent
- ☐ *Not applicable*

27. Are the help and the treatment you are receiving at the institution giving you confidence that life will be better after discharge?

- ☐ Not at all
- ☐ To a small extent
- ☐ To some extent
- ☐ To a large extent
- ☐ To a very large extent
- ☐ *Not applicable*

28. Overall, have the help and the treatment you have received at the institution been satisfactory?

- ☐ Not at all
- ☐ To a small extent
- ☐ To some extent
- ☐ To a large extent
- ☐ To a very large extent

Other assessments

29. Overall, to what extent have you benefitted from the treatment at the institution?

- ☐ No benefit
- ☐ Small benefit
- ☐ Some benefit
- ☐ Large benefit
- ☐ Very large benefit

Follow-up of physical health

30. Has your physical health been examined during this stay (e.g. blood tests, blood pressure, heart rate and weight)?

- ☐ Yes
- ☐ No
- ☐ *Not applicable*

31. Has the institution given you the opportunity to be physically active during admission (e.g. walking, jogging, exercise)?

- ☐ Yes
- ☐ No
- ☐ *Not applicable*

Help from the municipality

32. Overall, has the help you have received from the municipality where you live been satisfactory?

- ☐ Not at all
- ☐ To a small extent
- ☐ To some extent
- ☐ To a large extent
- ☐ To a very large extent
- ☐ *Have not received help*
- ☐ *Not applicable*

Overall assessment of the health services

When answering question 33 and 34, please consider all health services you have been in contact with, not just this institution.

33. Overall, what do you think of the help you have received from the health services for your mental health issues?

- ☐ Very poor
- ☐ Quite poor
- ☐ Neither poor nor good
- ☐ Quite good
- ☐ Very good

34. Do you find that the different health services have collaborated well in order to help you with your mental health issues?

- ☐ Not at all
- ☐ To a small extent
- ☐ To some extent
- ☐ To a large extent
- ☐ To a very large extent
- ☐ *Don't know*
- ☐ *Not applicable*

A little about you and your background

35. How would you describe your *mental* health?

- ☐ Very poor
- ☐ Quite poor
- ☐ Neither poor nor good
- ☐ Quite good
- ☐ Very good

A little about you and your background

36. Overall, how are you feeling today?

- ☐ Very poor
- ☐ Quite poor
- ☐ Neither poor nor good
- ☐ Quite good
- ☐ Very good

37. How would you describe your *physical* health?

- ☐ Excellent
- ☐ Very good
- ☐ Good
- ☐ Fair
- ☐ Poor

38. What is the main reason for your admission? *Enter one X only.*

- ☐ Eating disorder
- ☐ Substance abuse or dependency problem
- ☐ Anxiety and/or depression
- ☐ Psychosis/schizophrenia
- ☐ Other

39. Have you experienced coercion during this stay (e.g. involuntary admission, forced medication or being restrained)?

- ☐ Yes >>> go to question 40
- ☐ No >>> go to question 41

40. If you have experienced coercion, have you been informed that a decision has been made to allow use of coercion in your case?

- ☐ Yes
- ☐ No

41. Have you been admitted to a psychiatric institution prior to this admission?

- ☐ No
- ☐ Yes, once
- ☐ Yes, 2 times
- ☐ Yes, 3-5 times
- ☐ Yes, more than 5 times

42. Are you female or male?

- ☐ Female
- ☐ Male

43. How old are you?

- ☐ 18-24
- ☐ 25-44
- ☐ 45-66
- ☐ 67 or older

44. Are you married/living with a partner?

- ☐ Yes
- ☐ No

45. What is your highest level of education?

- ☐ Compulsory primary school (grades 1-10)
- ☐ Upper secondary school
- ☐ College/university

46. Where were you born?

- ☐ Norway
- ☐ In a Nordic country (other than Norway)
- ☐ Western Europe (other than a Nordic country)
- ☐ EU country in Eastern Europe
- ☐ Eastern Europe (not a country in the EU)
- ☐ Africa
- ☐ Asia (including Turkey)
- ☐ North America
- ☐ South America or Central America
- ☐ Oceania

Feel free to write more about your experiences with your stay here:

Thank you for taking the time to complete the survey!

NIPH
Norwegian Institute of Public Health
